# Human placental trophoblast invasion and differentiation: a particular focus on Wnt signaling

**DOI:** 10.3389/fgene.2013.00190

**Published:** 2013-09-26

**Authors:** Martin Knöfler, Jürgen Pollheimer

**Affiliations:** Department of Obstetrics and Fetal-Maternal Medicine, Reproductive Biology Unit, Medical University of ViennaAustria

**Keywords:** placenta, human, trophoblast, invasion, Wnt

## Abstract

Wingless ligands, a family of secreted proteins, are critically involved in organ development and tissue homeostasis by ensuring balanced rates of stem cell proliferation, cell death and differentiation. Wnt signaling components also play crucial roles in murine placental development controlling trophoblast lineage determination, chorioallantoic fusion and placental branching morphogenesis. However, the role of the pathway in human placentation, trophoblast development and differentiation is only partly understood. Here, we summarize our present knowledge about Wnt signaling in the human placenta and discuss its potential role in physiological and aberrant trophoblast invasion, gestational diseases and choriocarcinoma formation. Differentiation of proliferative first trimester cytotrophoblasts into invasive extravillous trophoblasts is associated with nuclear recruitment of β -catenin and induction of Wnt-dependent T-cell factor 4 suggesting that canonical Wnt signaling could be important for the formation and function of extravillous trophoblasts. Indeed, activation of the pathway was shown to promote trophoblast invasion in different *in vitro* trophoblast model systems as well as trophoblast cell fusion. Methylation-mediated silencing of inhibitors of Wnt signaling provided evidence for epigenetic activation of the pathway in placental tissues and choriocarcinoma cells. Similarly, abundant nuclear expression of β -catenin in invasive trophoblasts of complete hydatidiform moles suggested a role for hyper-activated Wnt signaling. In contrast, upregulation of Wnt inhibitors was noticed in placentae of women with preeclampsia, a disease characterized by shallow trophoblast invasion and incomplete spiral artery remodeling. Moreover, changes in Wnt signaling have been observed upon cytomegalovirus infection and in recurrent abortions. In summary, the current literature suggests a critical role of Wnt signaling in physiological and abnormal trophoblast function.

## DEVELOPMENT AND FUNCTION OF THE HUMAN PLACENTA

**FIGURE 1:**
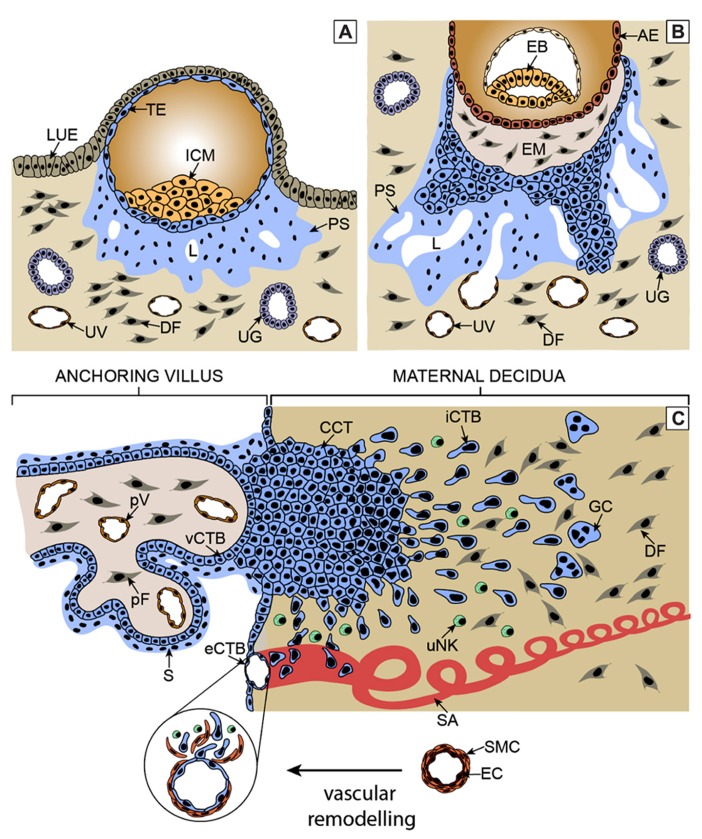
**Critical steps of human placental development. (A)** After implantation stems cells of the trophectoderm give rise to the primitive syncytium by cell fusion. In this region lacunae, the ancestor of the intervillous space, are formed. Some of the lacunae erode uterine vessels. **(B)** At a subsequent stage, proliferative cytotrophoblasts (CTBs) emanate from the trophectoderm, break through the primitive syncytium and contact the basal plate thereby forming primary villi. **(C)** Tertiary villi are built upon migration of extraembryonic mesodermal cells into the primary structures and vascularization. At distal sites, proliferative cell columns are formed which give rise to different invasive extravillous trophoblast subtypes. iCTBs migrate into decidual stroma approach vessels from outside and eventually form giant cells as the end stage of the invasive differentiation pathway. Endovascular trophoblasts migrate into spiral arteries and contribute to uNK cell-initiated remodeling within the decidua and the upper part of the myometrium. AE, amniotic epithelium; CCT, cell column trophoblast; DF, decidual fibroblast; EB, embryoblast; EM, extraembryonic mesoderm; eCTB, endovascular cytotrophoblast; GC, giant cell; ICM, inner cell mass, iCTB, interstitial cytotrophoblast; LUE, luminal uterine epithelium; L, lacunae, pF, placental fibroblast; PS, primitive syncytium; pV, placental vessel; SA, spiral artery; S, syncytium; TE, trophectoderm; UG, uterine gland; uNK, uterine NK cell; UV, uterine vessel; vCTB, villous cytotrophoblast.

Development of the human placenta is critical for embryonic development and successful pregnancy outcome. Immediately after implantation, trophectodermal cells forming the outermost epithelial layer of the blastocyst give rise to diverse trophoblast cell types (Hamilton and Boyd, 1960; Cross et al., 1994). Cell fusion generates the primitive syncytium underneath the implanted embryo, which may represent the earliest invasive trophoblast cell type migrating into the maternal endometrium (**Figure 1A**). After formation of the lacuna system, the ancestor of the intervillous space, cytotrophoblasts (CTBs) emanating from the trophectodermal layer generate primary villi by proliferation and invasion through the primitive syncytium (**Figure 1B**). Throughout pregnancy, these primary villi transform into secondary and tertiary villi characterized by invasion of extraembryonic mesenchymal cells, villous branching, and vascularization. During the first trimester of pregnancy two types of mature villi can be discriminated, which are floating and anchoring villi (**Figure 1C**). Floating villi connected to the intervillous space represent the transport units of human placenta. After establishment of blood flow nutrients and oxygen pass the epithelial layers of these villi ensuring appropriate fetal development and growth. Multinucleated syncytiotrophoblasts covering the surface of floating villi are continuously generated by asymmetrical cell division, differentiation and fusion of villous cytotrophoblasts (vCTBs) with the developing syncytium (Aplin, 2010). The latter also secretes numerous hormones, such as human chorionic gonadotrophin, into the maternal circulation, which are required for maintenance and immunological adaptation of pregnancy (Bansal et al., 2012). Fusion process and numbers of vCTBs decrease during pregnancy. Hence at term, syncytiotrophoblasts are in close contact with placental vessels allowing efficient nutrient uptake by the fetus.

Villi connected to the basal plate of the human placenta give rise to proliferative cell columns from which differentiated extravillous trophoblast (EVT) cell types are generated. At early stages of pregnancy, invasive endovascular cytotrophoblasts (eCTBs) plug the maternal arterioles to prevent premature onset of blood flow into the intervillous space (Pijnenborg et al., 2006). Failures in this process were shown to be associated with pregnancy complications such as abortions likely due to the premature rise in oxygen levels which may provoke oxidative stress and damage of placental villi (Hustin et al., 1990; Burton et al., 2010). Besides endovascular invasion, interstitial cytotrophoblasts (iCTBs) migrate into the maternal decidua likely cross-talking to diverse uterine cell types, including uterine natural killer cells, macrophages, and decidual stromal cells (Bulmer et al., 2010; Oreshkova et al., 2012). These interactions are thought to be important for immunological acceptance of the placental/fetal allograft as well as for the timing and depth of trophoblast invasion (Redman and Sargent, 2010). In particular, interaction of paternal human leukocyte antigen (HLA)-C expressed on iCTBs with maternal killer cell immunoglobulin-like receptors (KIR) are thought to play a pivotal role in placentation and reproductive success (Hiby et al., 2004). In the absence of blood flow, trophoblast invasion might be controlled by factors secreted from uterine glands, such as epidermal growth factor (EGF) and vascular endothelial growth factor (vEGF), which are also thought to be crucial for placental and embryonic growth at this early stage of pregnancy (Burton et al., 2007). With the establishment of the maternal-placental circulation, the placenta switches from histiotrophic to haemotrophic nutrition around the 12th week of gestation, plugging of vessels is dissolved and extensive remodeling occurs. Transformation of the maternal spiral arteries into large diameter vessels likely ensures adapted nutrient supply, reduced vessel contractility and constant oxygen delivery at low blood pressure to the developing fetus (Burton et al., 2010). Vessel remodeling might be initiated by uterine NK cells and both types of differentiated EVTs are thought to play a crucial role in completing this process (Robson et al., 2012). Whereas eCTBs displace maternal endothelial cells and remodel decidual and superficial myometrial spiral arteries, iCTBs approach the vessels from outside and contribute to elastolysis and disruption of the vascular wall. This involves a series of events such as trophoblast-induced apoptosis of vascular smooth muscle cells (Harris, 2011). Failures in EVT invasion, remodeling and CTB gene expression were noticed in different pregnancy diseases such as preeclampsia or severe intrauterine growth restriction (Pijnenborg et al., 1991, 2006; Zhou et al., 2013). Abnormal contractility and pressure may provoke hypoxia re-oxygenation injuries of floating villi thereby inducing secretion of adverse cytokines and antiangiogenic molecules such as soluble Fms-like tyrosine kinase-1 (sFLT-1) and elevated shedding of syncytiotrophoblast microparticles (Burton et al., 2010; Redman et al., 2012; Tal, 2012). The latter are thought to contribute to maternal endothelial cell dysfunction as part of a global, systemic inflammatory response observed in preeclampsia.

## DIFFERENTIATION AND INVASION OF HUMAN EXTRAVILLOUS TROPHOBLAST

Differentiation of proliferative CTBs into growth-arrested EVTs, invading decidual tissue and vessels, is thought to involve a series of well-controlled molecular steps which, however, are poorly understood. This is partly due to the fact that EVT differentiation *in vitro* can only be studied upon access to first trimester placental samples, which in general is limited due to ethical considerations. Growth of the trophoblast cell column harboring progenitor cells for the invasive differentiation pathway may involve paracrine factors released from the underlying placental mesenchyme such as IGF molecules which also promote proliferation of vCTBs *in vitro* (Aplin et al., 2000; Forbes et al., 2008). Since spontaneous outgrowth and migration in villous explant cultures is achieved in the absence of serum, it is likely that the intrinsic molecular program of the placental villus is also sufficient for the particular differentiation process *in vivo*. However, the precise mechanisms controlling integrity and stability of cell columns allowing for balanced rates of growth and differentiation have not yet been elucidated. Adhesive interactions between L-selectin expressed in cell columns and its carbohydrate ligands could play a role (Prakobphol et al., 2006). Moreover, different transcription factors such as oxygen-dependent hypoxia-inducible factor 1α (HIF1α ) and Stox1, discussed elsewhere in this issue, are critical for trophoblast cell proliferation and inhibit differentiation into EVTs (Caniggia et al., 2000b; van Dijk and Oudejans, 2011). In contrast, AP-2α , signal transducer and activator of transcription 3 (STAT3) or glial cells missing 1 (GCM1) promote trophoblast invasion and GCM1 was also shown to inhibit proliferation of vCTBs (Poehlmann et al., 2005; Baczyk et al., 2009; Biadasiewicz et al., 2011). Hence, it is assumed that a set of key regulatory transcription factors controls the switch between trophoblast proliferation and EVT differentiation (Loregger et al., 2003; Knöfler and Pollheimer, 2012). EVT formation is accompanied by the expression of distinct intergrins which are induced in a distance-dependent manner *in vivo* as well as *in vitro* (Damsky et al., 1994; Aplin et al., 1999). Again, the molecular basis for differentiation-dependent integrin switching remains unknown. Increasing oxygen concentrations during pregnancy and contact with the decidual matrix likely play major roles. Interestingly, accumulating evidence suggests that failures in EVT differentiation could contribute to the pathogenesis of pregnancy diseases with restricted trophoblast invasion and remodeling. Expression of inhibitor of DNA binding 2 (Id2), blocking the binding activity of differentiation-promoting basic helix-loop-helix (bHLH) proteins through heterodimerisation, was shown to be downregulated in EVTs of normal pregnancy but maintained in preeclamptic placental tissue (Janatpour et al., 2000). Along those lines, inhibition of HIF1α -dependent TGFβ3, acting as a negative regulator of trophoblast invasion, restored migration in explant cultures of preeclamptic villi emphasizing the particular role of oxygen in EVT differentiation (Caniggia et al., 1999, 2000a). Moreover, upregulation of EVT-specific genes and invasion were impaired in trophoblasts isolated from preeclamptic placentae (Lim et al., 1997). Of importance, eCTBs express a characteristic pattern of vascular adhesion molecules which, however, is abnormal in preeclamptic tissues (Zhou et al., 1997a).

While the hierarchy and cross-talk of critical molecular events controlling EVT differentiation await further investigations, regulation of trophoblast invasion has been investigated in a vast number of studies using primary cells, choriocarcinoma cells and established non-tumorigenic trophoblast cell lines. The different invasive trophoblast cell types produce sets of proteases, i.e., matrix metalloproteinases (MMP), urokinase plasminogen activator (uPA) and cathepsins, which are thought to degrade decidual extracellular matrix proteins and thereby facilitate cell invasiveness. The respective inhibitors, tissue inhibitors of metalloproteinases (TIMPs) and plasminogen activator inhibitors (PAIs) are produced by EVTs as well as decidual cells to limit the extent of trophoblast invasion. Numerous soluble factors expressed at the fetal-maternal interface including chemokines, cytokines and angiogenic factors were shown to promote trophoblast motility in an autocrine or paracrine manner (Bischof et al., 2000; Lala and Chakraborty, 2003; Knöfler, 2010). As a common theme, the secreted proteins were shown to stimulate MMP expression and secretion, in particular the gelatinases MMP-2 and MMP-9. Inhibitory proteins such as TNF, Nodal or TGFβ could restrain trophoblast motility by increasing expression of TIMPs and PAIs (Lala and Graham, 1990; Haider and Knöfler, 2009; Nadeem et al., 2011). Although a complex interplay of growth factors likely controls trophoblast cell migration and invasion, it remains unclear whether all of the currently identified effects truly play a role *in vivo*. Tumorigenic and non-tumorigenic cell types as well as hybridomas used in functional studies may not accurately mimic trophoblast cell behavior. Indeed, overall gene expression profiles of primary CTBs and EVT cultures differ considerably from the different established trophoblast cell lines (Bilban et al., 2010). Moreover, compared to primary cells, a diverging HLA profile was identified in the immortalized trophoblast cell lines. Villous CTBs lack surface expression of classical HLA molecules, but EVTs produce HLA-C, -E, and -G upon differentiation. JEG-3 choriocarcinoma cells show a similar HLA profile as EVTs, whereas several immortalized cell lines produce HLA-A and -B, suggesting abnormal activation of these genes during the immortalization procedure or a non-trophoblastic origin of these cells (Apps et al., 2009).

Furthermore, published literature suggests key signaling pathways that are involved in trophoblast motility. Abundant growth factors such as hCG, EGF, HGF, or IGF2 activate MAPK kinase (MEK)/extracellular regulated kinase (ERK) and phosphoinositide 3-kinase (PI3K)/AKT/mammalian target of rapamycin (mTOR) signaling, whereas prostaglandins were shown to act through the Rho-Rock pathway (Pollheimer and Knöfler, 2005; Knöfler, 2010). Besides expression of TIMPs and PAIs, downregulation of signaling kinase activity could represent a mechanism to limit the extent of trophoblast invasion. For example endostatin, which could be released from decidual collagen XVIII by EVT-mediated proteolytic cleavage, was shown to impair growth factor-induced AKT/mTOR phosphorylation and cell migration (Pollheimer et al., 2004, 2005, 2011).

To identify novel genes and pathways controlling trophoblast motility and differentiation we and others recently performed comparative gene expression studies of CTBs and EVTs isolated from first trimester placental tissues. Chip-based profiling of EGFR-positive CTBs, isolated by flow cytometry, and EVTs, generated by seeding of the CTBs on fibronectin for 12 h, resulted in the identification of 3433 mRNAs which are at least two-fold differentially expressed between the two cell populations (Apps et al., 2011). Using immunopurified CTBs and EVTs isolated from outgrowths of villous explant cultures and gene chips with a lower number of probe sets compared to the aforementioned study, we detected 991 differentially expressed transcripts in our analyses (Bilban et al., 2009). One of these mRNAs which was found to be induced upon EVT differentiation encoded TCF-4, one of the key transcription factors in Wnt signaling (Roose and Clevers, 1999). Hence, this result prompted us to investigate the expression pattern of Wnt signaling components and the general role of the canonical signaling pathway in human trophoblast migration and invasion.

## Wnt SIGNALING PATHWAYS

Besides Hippo, Hedgehog, Notch and TGFβ signaling, Wnt signaling represents one of the few conserved pathways critically involved in developmental processes. From *Drosophila* to human, the particular signal transduction cascade controls early axis formation, limb patterning and organogenesis (Logan and Nusse, 2004; Clevers, 2006). In adults, Wnt controls homeostasis of regenerating tissues such by regulating stem cell maintenance, cell fate decisions and differentiation (Clevers, 2006; Herr et al., 2012). Abnormal Wnt signaling has been described in a variety of human diseases including different cancers, diabetes or neurodegenerative disorders (Polakis, 2000; Al-Harthi, 2012). Wnts comprise a family of palmitoylated, cysteine-rich glycoproteins, which due to their low solubility are secreted in a lipoprotein-bound form or through exosomes (Herr et al., 2012). The first described member of this family of secreted ligands was the Wnt1 proto-oncogene, which is homologous to the *Drosophila* gene Wingless. Originally, the gene has been named int-1 since it has been identified as an integration site for the murine mammary tumor virus which can provoke breast cancer (Nusse et al., 1991). In humans, 19 different Wnt ligands and 10 seven-transmembrane domain frizzled (Fzd) receptors have been identified (Wodarz and Nusse, 1998). The latter interact with low density lipoprotein receptor related proteins (LRP-5 or -6) forming a functional, heterodimeric receptor for canonical Wnt signaling. It is likely that the complex interplay of different Wnts with Fzds provokes specific Wnt responses depending on the receptor context and the particular cell type. Stabilization and nuclear recruitment of β -catenin is a hallmark of the canonical pathway. However, Wnt ligands also trigger non-canonical, β -catenin-independent signaling including the Wnt/Ca^2+^ and the Wnt/planar cell polarity (PCP) pathway (Gordon and Nusse, 2006; Hendrickx and Leyns, 2008; van Amerongen and Nusse, 2009).

### CANONICAL Wnt SIGNALING

Canonical Wnt signaling involves a series of steps resulting in the stabilization and nuclear translocation of β -catenin (Gordon and Nusse, 2006). In unstimulated cells, β -catenin is predominantly found at adherens junctions where it binds to E-cadherin and α -catenin and thereby maintains epithelial structure and polarity. Cytosolic levels of β -catenin are low since it is degraded in a destruction complex consisting of adenomatous polyposis coli (APC), Axin, casein kinase Iα (CKIα ) and glycogen synthase kinase 3β (GSK-3β ). The latter phosphorylates β -catenin at its N-terminus and thereby induces binding of β -transducin repeat-containing protein (β -TrCP) and its associated E3 ubiquitin ligase. This results in ubiquitination and proteasomal degradation of β -catenin (Stamos and Weis, 2013). In contrast, Wnt stimulation promotes Fzd-LRP heterodimerisation and cytosolic stabilization of β -catenin by disruption of the APC/Axin/GSK-3β /CK1α destruction complex. Upon binding of Wnt to the cysteine-rich domain of Fzd, the multifunctional protein Disheveled (Dvl) is recruited to the cytosolic portion of the heterodimeric receptor and thereby provokes binding of Axin and GSK-3β as well as GSK-3β -mediated phosphorylation of LRP-5/6 (Mao et al., 2001; Bilic et al., 2007). This event could either inhibit the catalytic activity of GSK-3β toward β -catenin promoting sequestration or induce internalization and lysosomal degradation of components of the destruction complex (Metcalfe and Bienz, 2011). As a consequence cytoplasmic concentrations of β -catenin increase and active de-phosphorylated β -catenin translocates to the nucleus where it binds to transcription factors of the lymphoid enhancer factor-1 (LEF-1)/TCF family (Clevers, 2006; Gordon and Nusse, 2006). LEF/TCF proteins are high mobility group proteins lacking transcriptional activity and hence require co-activators or co-repressors for their function. Binding of β -catenin to LEF/TCF converts these proteins into transcriptional activators by displacing histone deacetylases (HDACs) and inhibitors of the Groucho protein family followed by recruitment of the Legless family docking protein Bcl9, CBP/p300 and histone acetylases (Gordon and Nusse, 2006; Archbold et al., 2012). Activation of LEF-1/TCF then provokes transcription of numerous genes controlling developmental processes, cell cycle, differentiation and cell invasion such as cyclin D1, c-myc, c-jun, MMPs, urokinase plasminogen activator receptor (uPAR), Notch signaling factors and many others depicted at the Wnt homepage^1^. Moreover, Wnt signaling components such as Axin, TCFs or Fzds are often controlled by a feedback loop upon Wnt activation. Accumulating evidence, however, suggests that our view of canonical Wnt signaling is still too simplistic. Proteins of the destruction complex can enter the nucleus and influence trafficking of Wnt signaling components as well as gene transcription. Numerous soluble negative regulators of Wnt signaling such as different Dickkopf (Dkk) or secreted frizzled-related proteins (sFRPs) binding to Wnts and LRP, respectively, have been identified (Gordon and Nusse, 2006). TCFs interact with several other regulatory transcription factors, for example Smads, c-jun or Cdx proteins, likely determining specificity of binding to Wnt response elements (Archbold et al., 2012). Moreover, TCF/β -catenin transcriptional complexes can also repress transcription and β -catenin can be recruited to other transcription factors than LEF/TCF, e.g., steroid hormone receptors, in a Wnt-dependent manner (Beildeck et al., 2010).

Several alterations in Wnt signaling components have been detected in cancer cells provoking nuclear accumulation of β -catenin and aberrant Wnt signaling (Camilli and Weeraratna, 2010). While mutations in Wnt ligands are rare, mutations in the APC tumor suppressor gene have been identified in the majority of sporadic colorectal cancers. Moreover, activating mutations in β -catenin inhibiting its GSK-3β -dependent degradation were detected in colon, prostate and other malign tumors. In addition, there is also compelling evidence that epigenetic changes of the Wnt pathway contribute to tumorigenesis since downregulation of sFRP gene transcription through promoter methylation has been observed in different epithelial cancers (Herr et al., 2012).

### NON-CANONICAL Wnt SIGNALING

The fact that different Wnts can exert effects on cells independently of β -catenin adds further complexity to the particular signaling pathway. Ligands such as Wnt5a or Wnt11 can activate the Wnt/PCP and the Wnt/Ca^2+^ pathway by binding to Fzds and activating Dvl independently of LRP-5 or -6 (Komiya and Habas, 2008). Induction of Wnt/PCP signaling, originally identified in different developmental processes of *Drosophila*, involves the G-proteins Rac and RhoA and the downstream effectors c-jun NH_2_-terminal kinase (JNK) and Rho-associated kinase (ROCK), respectively. Wnt/PCP signaling is critically involved in the formation of embryonic tissues and organs and aberrant activation of the pathway was shown to promote metastasis of different cancer types (Wang, 2009).

On the other hand, Wnt/Ca^2+^ signaling inhibits cGMP-dependent protein kinase (PKG), which blocks Ca^2+^ release in unstimulated cells and activates phospholipase C (PLC) and elevation of inositol 1,4,5-trisphosphate (IP_3_) thereby releasing Ca^2+^ from the endoplasmic reticulum (Kohn and Moon, 2005). Increased cytosolic Ca^2+^ levels finally stimulate activity of protein kinase C (PKC), calcium/calmodulin-dependent kinase II (CamKII) and calcineurin which provoke nuclear recruitment of nuclear factor κ B (NFκ B) and of nuclear factor of activated T cells (NF-AT) (Saneyoshi et al., 2002; Ma and Wang, 2006; De, 2011). In addition, Ca^2+^ accumulation upon Wnt5a stimulation can induce TGF-β -activated kinase (TAK1) and Nemo-like kinase (NEMO), which block TCF through phosphorylation and antagonize canonical Wnt signaling (Ishitani et al., 1999; Ishitani and Ishitani, 2013). Hence, in some cancers Wnt5a acts as a tumor suppressor. However, the effects of Wnt5a strongly depend on the receptor context. Binding to Fzd2, 3, 5, 6 induces Ca^2+^ signaling, but the ligand can also activate the canonical pathway upon interaction with Fzd4 and LRP (Mikels and Nusse, 2006; Nishita et al., 2010). Moreover, various studies suggest that different Wnts can interact with the receptor tyrosine kinases Ryk and Ror2 and promote developmental processes independently of Fzd or activate classical signaling pathways such as ERK or PI3K/AKT/mTOR (Yun et al., 2005; Kim et al., 2007; Fradkin et al., 2010; Minami et al., 2010). In conclusion, Wnts binding to canonical and diverse non-canonical receptors form a highly complex signaling network with a considerable overlap between the different pathways.

## Wnt SIGNALING IN PLACENTA AND TROPHOBLAST

As outlined above and discussed elsewhere, early placental development is associated with rapid generation of several trophoblast subtypes forming distinct functional villi in mice and men (Georgiades et al., 2002; Red-Horse et al., 2004). Likewise, the maternal uterus adapts to pregnancy by extensive tissue remodeling involving differentiation of stromal cells, angiogenesis and immunological alterations. These critical processes are initiated during the secretory phase of the menstrual cycle and upon implantation and early stages of placental development. Given the fact that Wnt signaling is important for organ development and tissue homeostasis, it may not be surprising that the pathway also has major roles in uterus formation, growth and differentiation. Gene targeting in mice revealed that β -catenin and different Wnts, such as Wnt4, Wnt5a, or Wnt7a, are critical for uterine development (Miller and Sassoon, 1998; Vainio et al., 1999; Mericskay et al., 2004; Arango et al., 2005). Wnt signaling components also play a role in stroma cell proliferation and differentiation for example Wnt4, Wnt6 (Li et al., 2013; Wang et al., 2013) or Dkk1, a progesterone-regulated gene which is induced in the endometrium upon decidualization (Tulac et al., 2006; Duncan et al., 2011).

This review focuses on function of Wnt signaling in trophoblast and placental development and differentiation, whereas a number of different papers summarize the role of canonical and non-canonical Wnt signaling in female reproductive tract development and differentiation, uterine function and decidualization (Chen et al., 2009; Sonderegger et al., 2010b; van der Horst et al., 2012; Wetendorf and DeMayo, 2012).

### Wnt SIGNALING IN MURINE PLACENTAL DEVELOPMENT

Expression of several Wnt ligands, Fzds and Dvl proteins has been noticed in the developing murine blastocyst during the pre-implantation period (Mohamed et al., 2004; Harwood et al., 2008). However, the canonical pathway does not seem to play a role in blastocyst formation. Embryos bearing homozygous deletion of β -catenin develop to the blastocyst stage but are affected upon gastrulation (Haegel et al., 1995). However, maternal β -catenin may have compensated the lack of embryonic β -catenin. Therefore, mothers harboring a conditional deletion of the gene in oocytes were additionally used in β -catenin knock-out studies (De Vries et al., 2004). Again, development into blastocysts was observed suggesting that β -catenin is not required for early pre-implantation development. Also, treatment with Dkk1 did not impair blastocyst formation (Xie et al., 2008). Non-canonical signaling such as the Wnt/Ca^2+^ pathway could be involved (Chen et al., 2009). On the other hand, there is evidence that the canonical Wnt pathway could regulate blastocyst development in other species for example in ruminants (Denicol et al., 2013).

Whereas the canonical Wnt pathway is dispensable for murine blastocyst development, it is critical for blastocyst activation, adhesion and implantation. Inhibition of the pathway through Dkk1 or small molecular inhibitors decreased implantation, which was also shown to be associated with induction of canonical β -catenin and downregulation of non-canonical Wnt-RhoA signaling (Xie et al., 2008). Likewise, treatment with sFRP2 was shown to decrease implantation rates (Mohamed et al., 2005). Induction of the canonical pathway in the uterine epithelium and myometrial smooth muscle cells at the site of implantation through trophectoderm-derived Wnt ligands seems to be important (Mohamed et al., 2005).

Wnt signaling could also play a key role during early trophoblast development in mice. Treatment of embryonic stem cells with Wnt3a induced formation of trophectodermal stem cells with the capacity to differentiate into spongiotrophoblasts and giant cells which was mediated through LEF-1-dependent induction of Cdx2, a key regulator of early trophoblast lineage determination (Chawengsaksophak et al., 2004; He et al., 2008).

Furthermore, gene targeting in mice provided evidence that Wnt signaling components control sequential steps of placental development. In particular, chorioallantoic fusion, branching morphogenesis, labyrinth development and placental angiogenesis were affected in these mutants (Cross et al., 2006). For example, gene knock-outs of Wnt7b, R-spondin3, a soluble activator of canonical Wnt signaling, or of both TCF-1 and LEF-1 show defects in chorioallantoic fusion thereby altering normal labyrinth development and function at later stages of embryogenesis (Galceran et al., 1999; Parr et al., 2001; Aoki et al., 2007). Similar to the above mentioned mutants, homozygous deletion of Wnt2 and Fzd5 affected branching and labyrinth formation, however, without alterations in chorioallantoic fusion (Monkley et al., 1996; Ishikawa et al., 2001). Moreover, placentae lacking Bcl-9, one of the co-activators of LEF-1/TCF, showed defective branchpoint initiation and a decrease in syncytiotrophoblast formation (Matsuura et al., 2011). GCM1, the key transcription factor in branching morphogenesis and trophoblast cell fusion (Anson-Cartwright et al., 2000), is probably the most critical Wnt target in mouse placental development since diminished expression of GCM1 was noticed in R-spondin3 and Bcl-9-mutant placentae (Aoki et al., 2007; Matsuura et al., 2011). Indeed, a recent study indicated that a positive feedback loop of Fzd5 and GCM1 controls different steps of placental morphogenesis promoting branchpoint initiation, chorionic trophoblast-specific vEGF expression and trophoblast syncytialisation (Lu et al., 2013). Placental phenotypes of mice with homozygous deletions of Wnt signaling genes are summarized in **Table 1**. Early stages of murine trophoblast invasion, however, might be negatively affected by canonical Wnt signaling since recombinant Dkk1 was shown to increase motility in co-cultivations of ectoplacental cones with decidual cells (Peng et al., 2008).

**Table 1:** Placental phenotypes of mice with homozygous deletion of Wnt signaling components.

Gene knock-out	Phenotype	Reference
Wnt2	Defects in labyrinthine zone	Monkley etal. (1996)
	Decreased number of fetal capillaries	
	Oedema formation	
	Fibrinoid deposition	
Wnt7b	Defect in alpha4-mediated chorioallantoic fusion	Parr etal. (2001)
	Disorganization of chorionic plate	
R-spondin3	Defects in chorioallantoic fusion, branching and labyrinth formation	Aoki etal. (2007)
	Reduced expression of Gcm1	
Fzd5	Defects in yolk sac angiogenesis and placental vasculogenesis	Ishikawa et al. (2001); Lu et al. (2013)
	Defective chorioallantoic branching	
	Decreased GCM1 expression	
Bcl9	Defective branching initiation	Matsuura etal. (2011)
	Failures in trophoblast syncytialisation	
LEF-1/TCF-1	Defects in chorioallantoic fusion	Galceran etal. (1999)

### EXPRESSION PATTERNS OF Wnt LIGANDS AND FRIZZLED RECEPTORS IN HUMAN TROPHOBLASTS

**FIGURE 2:**
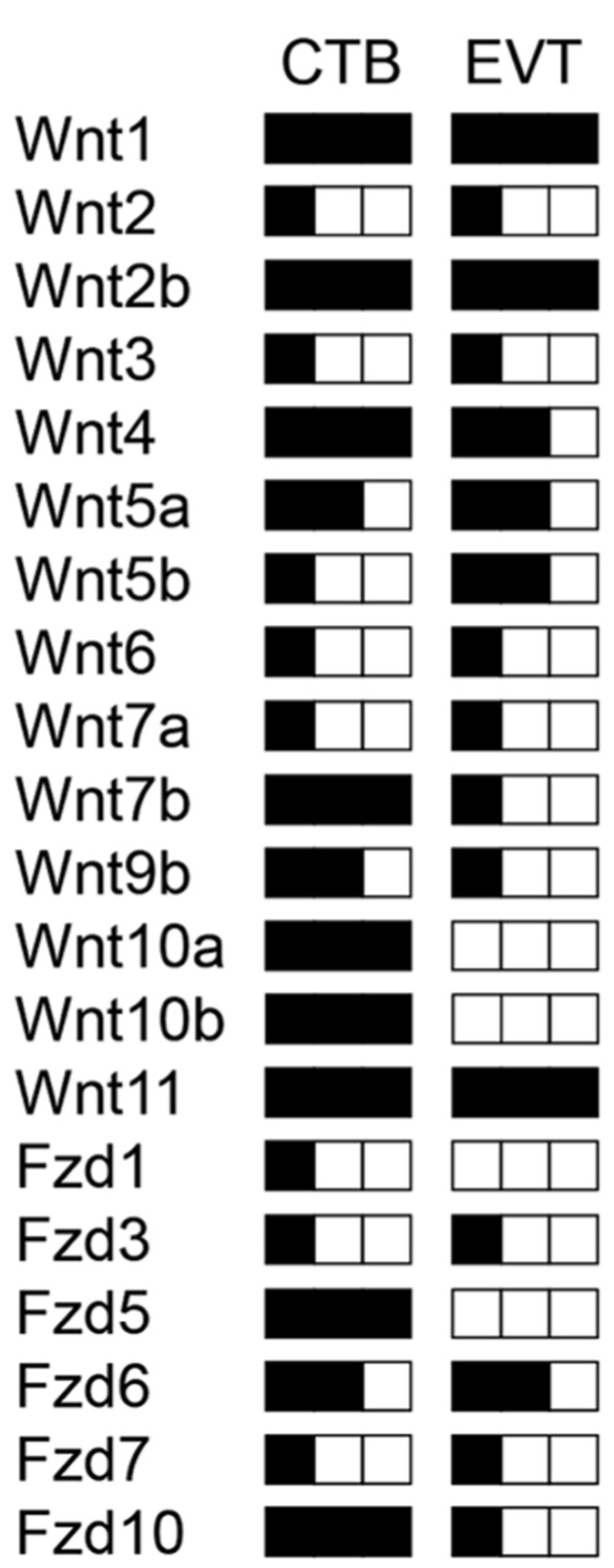
**Schematic illustration of transcript levels encoding Wnt ligands and Fzd receptors in isolated human first trimester CTBs and EVTs measured by semi-quantitative RT-PCR as published elsewhere (Sonderegger et al., 2007).** Low, medium and high levels of expression are indicated by one, two and three black squares, respectively.

Human trophoblast development is mostly studied in choriocarcinoma model systems and immortalized trophoblast cell lines, since primary placental material can only be obtained from very restricted time points during pregnancy. Trophoblast cell fusion can be investigated in isolated term CTBs involving numerous effectors (Morrish et al., 1998). Invasive properties of cells are lost at the end of pregnancy and therefore trophoblast motility and differentiation of progenitors into EVTs has to be analyzed in first trimester placentae using villous explant cultures and/or isolated CTBs (Pollheimer and Knöfler, 2005). Investigations in these primary cultures as well as in cell lines provided evidence for an autocrine role of Wnt signaling in human trophoblast proliferation and invasion (Sonderegger et al., 2010b). As a first step, our laboratory analyzed the expression patterns of all Wnt ligands and Fzd receptors in different trophoblast cell lines, isolated CTBs and total placental extracts of first and third trimester, as well as in villi and EVTs obtained from first trimester villous explant cultures using optimized, semi-quantitative RT-PCR (Sonderegger et al., 2007). 14 out of 19 Wnt ligands, and 8 out of 10 Fzd receptors were found to be expressed in total first trimester placenta. Most of these mRNAs were detectable in the villous trophoblast epithelium. In particular, abundantly (Wnt1, Wnt2b, Wnt4, Wnt7b, Wnt10a, Wnt10b, Wnt11), moderately (Wnt5a, Wnt9b) and lowly (Wnt2, Wnt3, Wnt5b, Wnt6, Wnt7a) -expressed Wnt ligands were present in first trimester CTBs as depicted (**Figure 2**). Hence, expression of canonical (for example Wnt1, Wnt2b, Wnt7b, Wnt10a, Wnt10b) as well as non-canonical Wnts (Wnt4, Wnt5a, Wnt11) suggests that different Wnt pathways may operate during human trophoblast development. Wnt1, Wnt7b, Wnt10a, and Wnt10b were downregulated from first trimester to term suggesting roles in trophoblasts of early pregnancy (Sonderegger et al., 2007). Whereas Fzd2 and Fzd4 are only produced in villous mesenchymal cells, Fzd1, Fzd3, Fzd5, Fzd6, Fzd7, and Fzd10 are expressed in CTBs. The latter was absent from villous fibroblasts suggesting a specific role in CTBs. Although none of the individual Wnts and Fzds have been studied in the context of human trophoblast invasion and differentiation so far, expression patterns and comparison to the situation in mouse placenta and other Wnt-dependent systems in reproduction allows to speculate about their potential roles.

**FIGURE 3:**
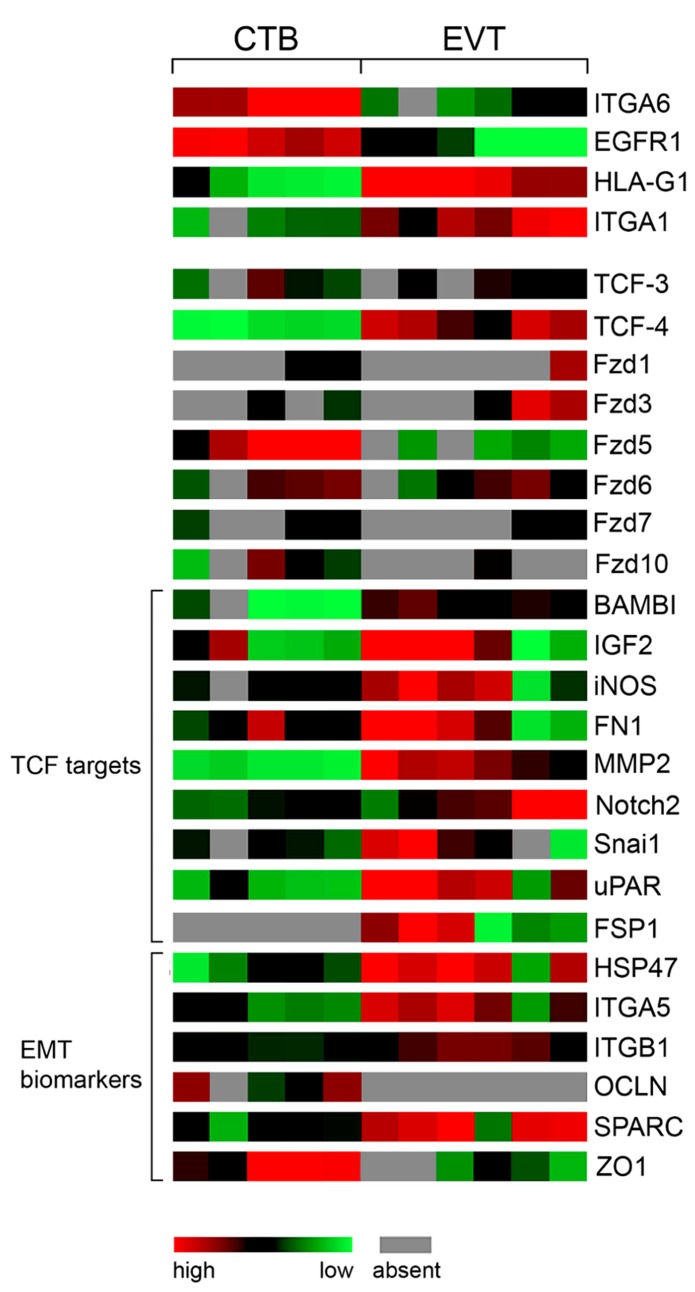
**Color-coded mRNA expression (GDS3523) in non-invasive CTBs (5 pools) and invasive EVTs (6 pools) analyzed by GEO DataSet Cluster Analysis online tool ().** Markers of EVT differentiation, Fzd receptors, putative TCF-4/β -catenin target genes as well as biomarkers of EMT are depicted.

Wnt4 is produced by CTBs as well as EVTs and could contribute to decidualization through the canonical pathway (Li et al., 2013). Wnt5a, secreted from trophoblast cell lines and primary cultures, may act through non-canonical pathways since it was unable to induce TCF/β -catenin-dependent transcription but antagonized the canonical pathway in trophoblasts (Sonderegger et al., 2007). Progesterone treatment of ovariectomized mice was shown to stimulate Wnt11 expression, another non-canonical Wnt ligand, whereas Wnt4 and Wnt7b are induced by estrogen which likely has implications for implantation (Hayashi et al., 2009) and decidualization as mentioned above. Interestingly, Wnt4, Wnt11 and Wnt7b are among the most abundantly produced Wnts in CTBs and Wnt7b expression decreased during EVT formation (**Figure 2**). Considering that Wnt7b is required for mouse placental development (Parr et al., 2001), the human homologue may also play a role in placenta formation and/or control critical functions during the first trimester of pregnancy such as trophoblast proliferation. Similarly, Wnt10a, Wnt10b, Fzd5, and Fzd10 were strongly expressed in CTBs but largely absent from EVTs or placental fibroblasts suggesting that these Wnt and Fzd members are also predominantly associated with early trophoblast cell growth. The principal Fzd receptor in EVTs is Fzd6, which operates through canonical as well as non-canonical pathways (Golan et al., 2004; Wu et al., 2009). Differential expression of Fzds between CTBs and EVTs is also detectable in our previously established gene expression profiles (Bilban et al., 2009) as shown in **Figure 3**. Whereas the role of Wnt10a, Wnt10b or FZD10 in murine placental development is unknown, Fzd5 is critical for branching morphogenesis as mentioned before (Lu et al., 2013). Hence, Fzd5 could also play a crucial role in human placental development. Furthermore, another semi-quantitative RT-PCR study suggested that several of the above mentioned Wnt ligands are regulated in a gestation-dependent manner in first trimester placenta (Grisaru-Granovsky et al., 2009) suggesting that specific combinations of Wnts could eventually control sequential steps of human trophoblast function and/or development. Moreover, the endometrial Wnt ligands Wnt2, Wnt4, Wnt5a, Wnt7a, Wnt8b, and Wnt3, the latter being regulated during the menstrual cycle, could affect trophoblast function in a paracrine manner (Tulac et al., 2003).

### Wnt SIGNALING IN HUMAN TROPHOBLAST FUNCTION AND DIFFERENTIATION

Studies in mice suggested that Wnt signaling is critical for activation and implantation of blastocysts (Mohamed et al., 2005). *In vitro* investigations with trophoblast cell lines and cultures also revealed a role of the particular signaling pathway in human trophoblast adhesion, invasion and differentiation.

Treatment of decidualized stromal cells with supernatants of trophoblasts provoked changes in the expression of some Wnt signaling components suggesting that soluble trophoblast-derived factors could influence endometrial function and differentiation through regulation of the Wnt pathway (Hess et al., 2007). Different Wnts secreted from EVTs could be the prime factors controlling expression of decidual Wnt signaling components since many of these genes are direct targets of TCF/β -catenin as summarized at the Wnt homepage^1^. Along those lines, attachment of spheroids prepared from JAR choriocarcinoma cells to endometrial Ishikawa cells was inhibited in the presence of Dkk1 (Liu et al., 2010). Similarly, 2,3,7,8-tetrachlorodibenzo-p-dioxin (TCDD), a negative effector of implantation, decreased attachment of JEG-3 or BeWo cell spheroids to different endometrial epithelial cell lines by suppressing β -catenin, which could be reverted upon treatment with a recombinant Wnt ligand (Tsang et al., 2012).

Moreover, expression analyses of TCFs in human first trimester placenta suggested that the canonical Wnt pathway is associated with the invasive differentiation process of trophoblasts (Pollheimer et al., 2006). Immunofluorescence of tissues revealed induction of TCF-4 protein in the nucleus of non-proliferating, p57/KIP2-positive EVTs as well as nuclear recruitment of β -catenin in a considerable number of these cells. Analyses of our expression profiles performed with total mRNA isolated from five and six different CTB and EVT cell preparations, respectively (Bilban et al., 2009), suggested induction of TCF-4 at the mRNA level (**Figure 3**). Moreover, stimulation with a Wnt ligand increased invasion of primary CTBs and trophoblastic SGHPL-5 cells in transwell assays and promoted migration in villous explant cultures seeded on collagen I, which was inhibited upon treatment with recombinant Dkk1 (Pollheimer et al., 2006; Sonderegger et al., 2010a). In addition, basal migration and invasion of the different trophoblast models were reduced in the presence of Dkk1 suggesting that the aforementioned canonical Wnts expressed in EVTs exert autocrine effects. Wnt stimulation was also shown to activate non-canonical AKT signaling and AKT-dependent motility of trophoblasts (Sonderegger et al., 2010a). Canonical LRP-5/6-FZD receptors were not involved since Wnt-dependent phosphorylation of AKT could not be inhibited upon supplementation of Dkk1. Also, cross-talk between the canonical Wnt pathway and AKT through AKT-induced phosphorylation and inactivation of GSK-3β as mentioned for other cells (Naito et al., 2005) has not been observed in trophoblasts since chemical AKT inhibitors did neither change nuclear accumulation of β -catenin nor the activity of a canonical Wnt reporter (Sonderegger et al., 2010a). One of the putative targets increasing invasiveness in a Wnt-dependent manner could be MMP-2, which is elevated in trophoblast supernatants upon Wnt stimulation and also has been described as a direct target of TCF/β -catenin (Wu et al., 2007; Sonderegger et al., 2010a). Although MMP-2 mRNA expression is associated with EVT differentiation (**Figure 3**), transcript levels did not change upon Wnt stimulation suggesting that the pathway induces MMP-2 secretion or affects its stability (Sonderegger et al., 2010a).

Although canonical Wnt signaling is strongly elevated upon EVT formation, CTBs also respond to Wnt signals. Wnt-dependent activation of TCF/β -catenin provoked increased proliferation and expression of the cell cycle regulator cyclin D1 as well as induction of Wnt signaling components (Pollheimer et al., 2006). Although TCF-4 is absent from proliferative CTBs, the canonical pathway might be activated through TCF-3 which is present in both CTBs and EVTs (**Figure 3**).

Furthermore, epigenetic analyses provided evidence for a general activation of Wnt signaling in placental tissues and isolated trophoblasts. Genes encoding negative regulators of the pathway, i.e., APC, sFRP2 and engrailed-1 were shown to be hypermethylated in trophoblasts, whereas these changes were not observed in placental fibroblast or leukocytes (Novakovic et al., 2008; Wong et al., 2008). This suggests that specific activation of the pathway in trophoblasts could play a role in placentation. In addition, other effectors than Wnt ligands likely contribute to stabilization of β -catenin and TCF/β -catenin-dependent trophoblast proliferation and invasion. Activation of protease activated receptor-1 (PAR1) provoked an increase in these processes whereas siRNA-mediated gene silencing of PAR1 or addition of soluble inhibitors downregulated TCF/β -catenin-induced proliferation and motility (Grisaru-Granovsky et al., 2009). Expression of StarD7, a member of the StAR1 lipid transfer proteins promoting proliferation and invasion of choriocarcinoma cells, was shown to be directly controlled by TCF/β -catenin (Rena et al., 2009; Flores-Martin et al., 2012).

In summary, the present literature implicates canonical Wnt signaling in human trophoblast proliferation and invasion. However, the question of whether TCF molecules are indeed regulators of EVT formation, in other words control the switch from proliferation to cell cycle arrest and differentiation is still unknown and currently under investigation in our laboratory. So far, canonical Wnt signaling has been identified as a regulator of trophoblast cell fusion. GCM1, the most critical factor in syncytialisation controlling expression of the fusogenic proteins syncytin-1 and -2, harbors TCF binding sites in one of its introns and silencing of TCF-4 or β -catenin impaired cAMP-induced cell fusion of BeWo choriocarcinoma cells (Matsuura et al., 2011). Moreover, Wnt targets such as Axin, BMP and activin membrane-bound inhibitor (BAMBI), and LEF-1 increased upon elevation of cAMP. However, it has to be mentioned that LEF-1 is only expressed in stromal cells of human placentae and TCF-3 and -4 are absent from villous CTBs after the 6th week of gestation (Pollheimer et al., 2006). Since trophoblast fusion occurs until the end of pregnancy, Wnt-dependent GCM1 expression and syncytialization may only operate during very early stages of human gestation. Similar to mice, Wnt signaling might also play a role in early human trophoblast lineage determination since the pathway was found to be activated upon BMP4-mediated differentiation of embryonic stem cells into trophoblasts (Marchand et al., 2011).

### Wnt TARGET GENES AND THE ROLE OF Wnt-DEPENDENT TRANSCRIPTION FACTORS IN EPITHELIAL TO MESENCHYMAL TRANSITION

Numerous studies indicated that cancer cell invasion shares several features with trophoblast invasion although the latter is precisely controlled in time and space. Besides expression of proteases, EVTs produce critical integrins such as the fibronectin receptor integrin α5β1 and the collagen/laminin receptor integrin α1β1 promoting trophoblast adhesion and migration (Damsky et al., 1994; Aplin et al., 1999). Conversely, EGF receptor 1 (EGFR1), indicative for the proliferative capacity of trophoblasts as well as integrin α6 (ITGA6), a marker of the polarized epithelium, were downregulated during EVT differentiation (Jokhi et al., 1994). Changes in the mRNA expression pattern of genes involved in EVT invasion and differentiation can also be monitored in our published chip data (Bilban et al., 2009) which are accessible via GEO profiles^2^. ITGA6 and EGFR are highly expressed in the five different CTB cell pools but weakly present in the six EVT preparations confirming the published literature (**Figure 3**). In contrast, HLA-G1, and the pro-migratory genes integrin α1 (ITGA1), integrin α5 (ITGA5) and fibronectin 1 (FN1) were upregulated in EVTs. Interestingly, various mRNAs such as BAMBI, a marker of metastasis in colon cancer (Fritzmann et al., 2009), insulin-like growth factor 2 (IGF2), inducible nitric oxide synthase (iNOS), fibronectin 1 (FN1), MMP-2, Notch2, uPAR, and Snai1 (also known as Snail1), which are all direct targets of TCF/β -catenin^1^, were found to be increased in the EVT pools concomitant with the upregulation of TCF-4 (**Figure 3**). Indeed, these genes have already been implicated in the control of trophoblast motility (Bischof et al., 2000; Lala and Chakraborty, 2003; Harris et al., 2008; Hunkapiller et al., 2011). Therefore, we speculate that nuclear recruitment of β -catenin and increased expression of TCF-4 in EVTs drives a set of genes promoting trophoblast invasion and migration.

Another molecular process critically involved in cancer cell invasion and metastasis is epithelial to mesenchymal transition (EMT), in which epithelial cells lose their polarity and gain fibroblast-like properties promoting invasion and migration (Zheng and Kang, 2013). Interestingly, EMT in cancer cells also provokes growth arrest and cells have to revert back to an epithelial phenotype (MET) allowing for cell growth and distant metastasis formation (Brabletz, 2012). Analyses of gene expression profiles suggest that EMT also occurs in invasive trophoblasts (**Figure 3**) which have stopped proliferation allowing for differentiation to take place. Although invasive trophoblasts do not induce mesenchymal vimentin, at least *in vitro*, and maintain expression of the epithelial marker cytokeratin 7, they upregulate typical EMT-associated mRNAs such as heat shock protein 47 (HSP47), Snail, ITGA5, ITGAB1, fibroblast-specific protein 1 (FSP1), MMP-2, and secreted protein acidic and rich in cysteine (SPARC) and downregulate genes associated with cell-cell adhesion and epithelial polarity such as the tight junction proteins occludin (OCLN) and ZO1. Also, transient loss of adherens junction proteins, i.e., membrane-bound β -catenin and E-cadherin, has been detected in the proximal invasion zone of anchoring villi using immunofluorescence in first trimester placental tissues (Zhou et al., 1997b). Elevated Wnt signaling, expression of LEF/TCF and nuclear recruitment of β -catenin is also a typical feature of EMT (Moustakas and Heldin, 2007). Hence, similar to cancer cells, induction of TCF-4 upon invasive trophoblast differentiation could orchestrate an EMT-like program to promote cell motility. In this process, TCF-4 could act as master regulator since it may not only directly control expression of pro-migratory EMT genes but also activate other critical key regulatory transcription factors inducing EMT such as Snail, Slug or ZEB1 (Medici et al., 2008; Sanchez-Tillo et al., 2011).

### Wnt signaling in gestational diseases

Changes in gene expression as well as in epigenetic modifications of Wnt signaling components were shown to be associated with different gestational diseases. Compared to normal tissues, higher numbers of β -catenin-positive EVT nuclei were detected in placentae of complete hydatidiform mole (CHM) suggesting that aberrant Wnt signaling could contribute to abnormal invasion in this pregnancy disorder (Pollheimer et al., 2006). Genes encoding APC and sFRP2 were shown to be hypermethylated in choriocarcinoma cells, indicating that inactivation of negative regulators of Wnt signaling likely contributes to the formation and/or progression of trophoblastic cancer cells (Novakovic et al., 2008; Wong et al., 2008). Similarly, Dkk1 was found to be absent from choriocarcinoma cells and re-expression of the gene induced growth arrest and apoptosis suggesting that the loss of Dkk1 is critical for tumor cell proliferation (Peng et al., 2006). In contrast, Dkk1 and sFRP4 were increased whereas Wnt2 and β -catenin were decreased in tissues of preeclamptic patients (Zhang et al., 2013a, b). Therefore, downregulation of the pathway could contribute to failed placentation and shallow trophoblast invasion observed in these pregnancies. Along those lines, elevated levels of Dkk1 were detected in women with recurrent abortions (Bao et al., 2013). Interestingly, cytomegalovirus (CMV) infection, a putative cause of spontaneous abortion and preterm delivery, was shown to decrease trophoblast proliferation and invasion involving inhibition of Wnt signaling. Infection of trophoblastic SGHPL-4 cells with the virus altered the localization of β -catenin and induced its degradation as well as downregulation of a canonical Wnt reporter (Angelova et al., 2012). Some of these effects might be exerted via peroxisome proliferator-activated receptor γ (PPAR- γ ) a negative regulator of trophoblast invasion, which is activated upon CMV infection and was shown to provoke proteasomal degradation of β -catenin in other cells (Liu et al., 2006; Rauwel et al., 2010).

## CONCLUSIONS

**FIGURE 4:**
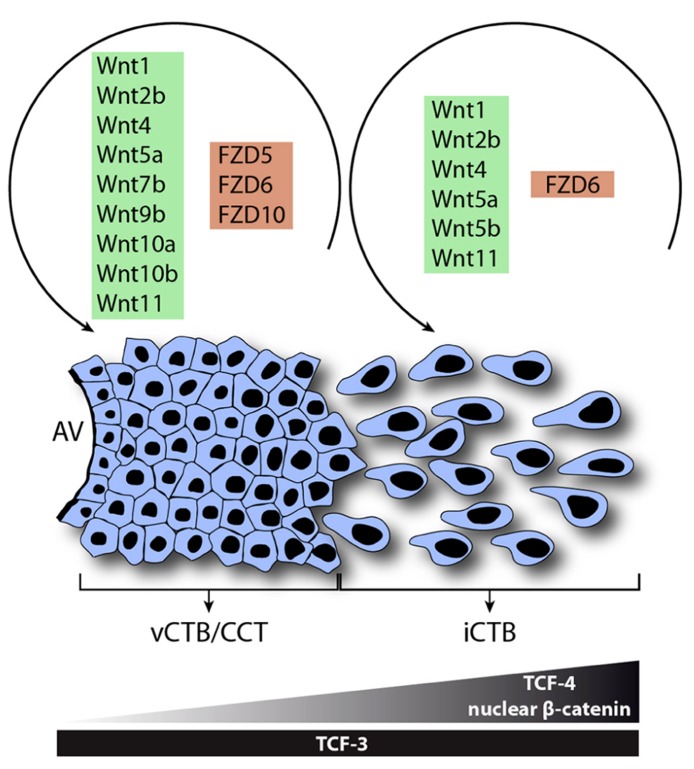
**Model system for the role of Wnt signaling in function and differentiation of the human anchoring villus.** Wnt ligands and Fzd receptors expressed in vCTBs/CCTs and EVTs are shown. During EVT formation, Wnt7b, Wnt9b, Wnt10a, Wnt10b, Fzd5, and Fzd10 are down-regulated suggesting a role in trophoblast proliferation. However, interstitial cytotrophoblasts (iCTBs) upregulate TCF-4 and nuclear β -catenin to promote trophoblast motility and possibly EVT differentiation.

In conclusion, the present literature suggests that Wnt signaling is critical for physiological processes of human trophoblasts. The pathway could play a role in blastocyst adhesion, implantation and early trophoblast lineage decisions. Moreover, Wnt signaling could regulate trophoblast cell fusion as well as development of the anchoring villus controlling proliferation, invasion and differentiation. In first trimester placenta numerous canonical and non-canonical Wnt ligands are expressed in CTBs and likely regulate proliferation or other functions of these cells through the most abundant receptors, i.e., Fzd5 and Fzd10 (**Figure 4**). Differentiation of CTBs into growth-arrested invasive EVTs is associated with increased expression of TCF-4, nuclear recruitment of β -catenin and elevated canonical Wnt activity controlling trophoblast motility potentially through Fzd6. Furthermore, TCF-4/β -catenin could activate a set of genes promoting invasion as well as the EMT-like features of migratory trophoblasts. Hence, Wnt signaling likely contributes to the strong, intrinsic differentiation program of EVTs and their inherently invasive properties. Hyperactivation of autocrine Wnt signaling could play a role in trophoblast disorders with elevated proliferation and invasion such as CHM and choriocarcinomas whereas downregulation of the pathway could be a cause of impaired placentation and trophoblast invasion observed in preeclampsia. Epigenetic changes such as methylation of negative regulators of Wnt signaling could contribute to the induction of Wnt signaling likely promoting normal placentation as well as trophoblast tumor progression. Besides autocrine control decidual Wnt ligands could modulate trophoblast function via canonical and/or non-canonical pathways. The fact that Dkk1 is expressed in the decidua and increased upon progesterone treatment could suggest a mechanism to restrain the extent of trophoblast invasion. Further studies are needed to delineate specific Wnt-Fzd interactions in human trophoblasts and to define the role of non-canonical Wnt signaling in normal and aberrant trophoblast proliferation, invasion and differentiation.

## Conflict of Interest Statement

The authors declare that the research was conducted in the absence of any commercial or financial relationships that could be construed as a potential conflict of interest.

## Footnotes

1

2

## References

[B1] Al-Harthi L. (2012). Wnt/beta-catenin and its diverse physiological cell signaling pathways in neurodegenerative and neuropsychiatric disorders. *J. Neuroimmune Pharmacol.* 7 725–730 10.1007/s11481-012-9412-x 23114888 PMC3690588

[B2] Angelova M. Zwezdaryk K. Ferris M. Shan B. Morris C. A. Sullivan D. E. (2012). Human cytomegalovirus infection dysregulates the canonical Wnt/beta-catenin signaling pathway. *PLoS Pathog.* 8:e1002959 10.1371/journal.ppat.1002959 PMC3469659 23071438

[B3] Anson-Cartwright L. Dawson K. Holmyard D. Fisher S. J. Lazzarini R. A. Cross J. C. (2000). The glial cells missing-1 protein is essential for branching morphogenesis in the chorioallantoic placenta. *Nat. Genet.* 25 311–314 10.1038/77076 10888880

[B4] Aoki M. Mieda M. Ikeda T. Hamada Y. Nakamura H. Okamoto H. (2007). R-spondin3 is required for mouse placental development. *Dev. Biol.* 301 218–226 10.1016/j.ydbio.2006.08.018 16963017

[B5] Aplin J. D. (2010). Developmental cell biology of human villous trophoblast: current research problems. *Int. J. Dev. Biol.* 54 323–329 10.1387/ijdb.082759ja 19876840

[B6] Aplin J. D. Haigh T. Jones C. J. Church H. J. Vicovac L. (1999). Development of cytotrophoblast columns from explanted first-trimester human placental villi: role of fibronectin and integrin alpha5beta1. *Biol. Reprod.* 60 828–838 10.1095/biolreprod60.4.828 10084955

[B7] Aplin J. D. Lacey H. Haigh T. Jones C. J. Chen C. P. Westwood M. (2000). Growth factor-extracellular matrix synergy in the control of trophoblast invasion. *Biochem. Soc. Trans.* 28 199–202 10816127 10.1042/bst0280199

[B8] Apps R. Murphy S. P. Fernando R. Gardner L. Ahad T. Moffett A. (2009). Human leucocyte antigen (HLA) expression of primary trophoblast cells and placental cell lines, determined using single antigen beads to characterize allotype specificities of anti-HLA antibodies. *Immunology* 127 26–39 10.1111/j.1365-2567.2008.03019.x 19368562 PMC2678179

[B9] Apps R. Sharkey A. Gardner L. Male V. Trotter M. Miller N. (2011). Genome-wide expression profile of first trimester villous and extravillous human trophoblast cells. *Placenta* 32 33–43 10.1016/j.placenta.2010.10.010 21075446 PMC3065343

[B10] Arango N. A. Szotek P. P. Manganaro T. F. Oliva E. Donahoe P. K. Teixeira J. (2005). Conditional deletion of beta-catenin in the mesenchyme of the developing mouse uterus results in a switch to adipogenesis in the myometrium. *Dev. Biol.* 288 276–283 10.1016/j.ydbio.2005.09.045 16256976

[B11] Archbold H. C. Yang Y. X. Chen L. Cadigan K. M. (2012). How do they do Wnt they do?: regulation of transcription by the Wnt/beta-catenin pathway. *Acta Physiol. (Oxf.)* 204 74–109 10.1111/j.1748-1716.2011.02293.x 21624092

[B12] Baczyk D. Drewlo S. Proctor L. Dunk C. Lye S. Kingdom J. (2009). Glial cell missing-1 transcription factor is required for the differentiation of the human trophoblast. *Cell Death Differ.* 16 719–727 10.1038/cdd.2009.1 19219068

[B13] Bansal A. S. Bora S. A. Saso S. Smith J. R. Johnson M. R. Thum M. Y. (2012). Mechanism of human chorionic gonadotrophin-mediated immunomodulation in pregnancy. *Expert Rev. Clin. Immunol.* 8 747–753 10.1586/eci.12.77 23167686

[B14] Bao S. H. Shuai W. Tong J. Wang L. Chen P. Duan T. (2013). Increased Dickkopf-1 expression in patients with unexplained recurrent spontaneous miscarriage. *Clin. Exp. Immunol.* 172 437–443 10.1111/cei.12066 23600832 PMC3646443

[B15] Beildeck M. E. Gelmann E. P. Byers S. W. (2010). Cross-regulation of signaling pathways: an example of nuclear hormone receptors and the canonical Wnt pathway. *Exp. Cell Res.* 316 1763–1772 10.1016/j.yexcr.2010.02.001 20138864 PMC2878914

[B16] Biadasiewicz K. Sonderegger S. Haslinger P. Haider S. Saleh L. Fiala C. (2011). Transcription factor AP-2alpha promotes EGF-dependent invasion of human trophoblast. *Endocrinology* 152 1458–1469 10.1210/en.2010-0936 21303946

[B17] Bilban M. Haslinger P. Prast J. Klinglmuller F. Woelfel T. Haider S. (2009). Identification of novel trophoblast invasion-related genes: heme oxygenase-1 controls motility via peroxisome proliferator-activated receptor gamma. *Endocrinology* 150 1000–1013 10.1210/en.2008-0456 18845641 PMC3064984

[B18] Bilban M. Tauber S. Haslinger P. Pollheimer J. Saleh L. Pehamberger H. (2010). Trophoblast invasion: assessment of cellular models using gene expression signatures. *Placenta* 31 989–996 10.1016/j.placenta.2010.08.011 20850871

[B19] Bilic J. Huang Y. L. Davidson G. Zimmermann T. Cruciat C. M. Bienz M. (2007). Wnt induces LRP6 signalosomes and promotes dishevelled-dependent LRP6 phosphorylation. *Science* 316 1619–1622 10.1126/science.1137065 17569865

[B20] Bischof P. Meisser A. Campana A. (2000). Paracrine and autocrine regulators of trophoblast invasion – a review. *Placenta *21(Suppl. A) S55–S60 10.1053/plac.2000.0521 10831123

[B21] Brabletz T. (2012). To differentiate or not – routes towards metastasis. *Nat. Rev. Cancer* 12 425–436 10.1038/nrc3265 22576165

[B22] Bulmer J. N. Williams P. J. Lash G. E. (2010). Immune cells in the placental bed. *Int. J. Dev. Biol.* 54 281–294 10.1387/ijdb.082763jb 19876837

[B23] Burton G. J. Jauniaux E. Charnock-Jones D. S. (2007). Human early placental development: potential roles of the endometrial glands. *Placenta* 28(Suppl. A) S64–S69 10.1016/j.placenta.2007.01.007 PMC1878510 17349689

[B24] Burton G. J. Jauniaux E. Charnock-Jones D. S. (2010). The influence of the intrauterine environment on human placental development. *Int. J. Dev. Biol.* 54 303–312 10.1387/ijdb.082764gb 19757391

[B25] Camilli T. C. Weeraratna A. T. (2010). Striking the target in Wnt-y conditions: intervening in Wnt signaling during cancer progression. *Biochem. Pharmacol.* 80 702–711 10.1016/j.bcp.2010.03.002 20211149 PMC2897923

[B26] Caniggia I. Grisaru-Gravnosky S. Kuliszewsky M. Post M. Lye S. J. (1999). Inhibition of TGF-beta 3 restores the invasive capability of extravillous trophoblasts in preeclamptic pregnancies. *J. Clin. Invest.* 103 1641–1650 10.1172/JCI6380 10377170 PMC408387

[B27] Caniggia I. Mostachfi H. Winter J. Gassmann M. Lye S. J. Kuliszewski M. (2000a). Hypoxia-inducible factor-1 mediates the biological effects of oxygen on human trophoblast differentiation through TGFbeta(3). *J. Clin. Invest.* 105 577–587 10.1172/JCI8316 10712429 PMC289179

[B28] Caniggia I. Winter J. Lye S. J. Post M. (2000b). Oxygen and placental development during the first trimester: implications for the pathophysiology of pre-eclampsia. *Placenta*21(Suppl. A) S25–S30 10.1053/plac.1999.0522 10831118

[B29] Chawengsaksophak K. De Graaff W. Rossant J. Deschamps J. Beck F. (2004). Cdx2 is essential for axial elongation in mouse development. *Proc. Natl. Acad. Sci. U.S.A.* 101 7641–7645 10.1073/pnas.0401654101 15136723 PMC419659

[B30] Chen Q. Zhang Y. Lu J. Wang Q. Wang S. Cao Y. (2009). Embryo-uterine cross-talk during implantation: the role of Wnt signaling. *Mol. Hum. Reprod.* 15 215–221 10.1093/molehr/gap009 19223336

[B31] Clevers H. (2006). Wnt/beta-catenin signaling in development and disease. *Cell* 127 469–480 10.1016/j.cell.2006.10.018 17081971

[B32] Cross J. C. Nakano H. Natale D. R. Simmons D. G. Watson E. D. (2006). Branching morphogenesis during development of placental villi. *Differentiation* 74 393–401 10.1111/j.1432-0436.2006.00103.x 16916377

[B33] Cross J. C. Werb Z. Fisher S. J. (1994). Implantation and the placenta: key pieces of the development puzzle. *Science* 266 1508–1518 10.1126/science.7985020 7985020

[B34] Damsky C. H. Librach C. Lim K. H. Fitzgerald M. L. Mcmaster M. T. Janatpour M. (1994). Integrin switching regulates normal trophoblast invasion. *Development* 120 3657–3666 7529679 10.1242/dev.120.12.3657

[B35] De A. (2011). Wnt/Ca2+ signaling pathway: a brief overview. *Acta Biochim. Biophys. Sin. (Shanghai)* 43 745–756 10.1093/abbs/gmr079 21903638

[B36] Denicol A. C. Dobbs K. B. Mclean K. M. Carambula S. F. Loureiro B. Hansen P. J. (2013). Canonical WNT signaling regulates development of bovine embryos to the blastocyst stage. *Sci. Rep.* 3 1266 10.1038/srep01266 PMC3569626 23405280

[B37] De Vries W. N. Evsikov A. V. Haac B. E. Fancher K. S. Holbrook A. E. Kemler R. (2004). Maternal beta-catenin and E-cadherin in mouse development. *Development* 131 4435–4445 10.1242/dev.01316 15306566

[B38] Duncan W. C. Shaw J. L. Burgess S. Mcdonald S. E. Critchley H. O. Horne A. W. (2011). Ectopic pregnancy as a model to identify endometrial genes and signaling pathways important in decidualization and regulated by local trophoblast. *PLoS ONE* 6:e23595 10.1371/journal.pone.0023595 PMC3157392 21858178

[B39] Flores-Martin J. Rena V. Marquez S. Panzetta-Dutari G. M. Genti-Raimondi S. (2012). StarD7 knockdown modulates ABCG2 expression, cell migration, proliferation, and differentiation of human choriocarcinoma JEG-3 cells. *PLoS ONE* 7:e44152 10.1371/journal.pone.0044152 PMC3430668 22952907

[B40] Forbes K. Westwood M. Baker P. N. Aplin J. D. (2008). Insulin-like growth factor I and II regulate the life cycle of trophoblast in the developing human placenta. *Am. J. Physiol. Cell Physiol.* 294 C1313–C1322 10.1152/ajpcell.00035.2008 18400990

[B41] Fradkin L. G. Dura J. M. Noordermeer J. N. (2010). Ryks: new partners for Wnts in the developing and regenerating nervous system. *Trends Neurosci.* 33 84–92 10.1016/j.tins.2009.11.005 20004982

[B42] Fritzmann J. Morkel M. Besser D. Budczies J. Kosel F. Brembeck F. H. (2009). A colorectal cancer expression profile that includes transforming growth factor beta inhibitor BAMBI predicts metastatic potential. *Gastroenterology* 137 165–175 10.1053/j.gastro.2009.03.041 19328798

[B43] Galceran J. Farinas I. Depew M. J. Clevers H. Grosschedl R. (1999). Wnt3a-/--like phenotype and limb deficiency in Lef1(-/-)Tcf1(-/-) mice. *Genes Dev.* 13 709–717 10.1101/gad.13.6.709 10090727 PMC316557

[B44] Georgiades P. Ferguson-Smith A. C. Burton G. J. (2002). Comparative developmental anatomy of the murine and human definitive placentae. *Placenta* 23 3–19 10.1053/plac.2001.0738 11869088

[B45] Golan T. Yaniv A. Bafico A. Liu G. Gazit A. (2004). The human Frizzled 6 (HFz6) acts as a negative regulator of the canonical Wnt. beta-catenin signaling cascade.* J. Biol. Chem.* 279 14879–14888 10.1074/jbc.M306421200 14747478

[B46] Gordon M. D. Nusse R. (2006). Wnt signaling: multiple pathways, multiple receptors, and multiple transcription factors. *J. Biol. Chem.* 281 22429–22433 10.1074/jbc.R600015200 16793760

[B47] Grisaru-Granovsky S. Maoz M. Barzilay O. Yin Y. J. Prus D. Bar-Shavit R. (2009). Protease activated receptor-1, PAR1, promotes placenta trophoblast invasion and beta-catenin stabilization. *J. Cell. Physiol.* 218 512–521 10.1002/jcp.21625 19040205

[B48] Haegel H. Larue L. Ohsugi M. Fedorov L. Herrenknecht K. Kemler R. (1995). Lack of beta-catenin affects mouse development at gastrulation. *Development* 121 3529–3537 8582267 10.1242/dev.121.11.3529

[B49] Haider S. Knöfler M. (2009). Human tumour necrosis factor: physiological and pathological roles in placenta and endometrium. *Placenta* 30 111–123 10.1016/j.placenta.2008.10.012 19027157 PMC2974215

[B50] Hamilton W. J. Boyd J. D. (1960). Development of the human placenta in the first three months of gestation. *J. Anat.* 94 297–328 14399291 PMC1244370

[B51] Harris L. K. (2011). IFPA gabor than award lecture: transformation of the spiral arteries in human pregnancy: key events in the remodelling timeline. *Placenta *32(Suppl. 2) S154–S158 10.1016/j.placenta.2010.11.018 21167598

[B52] Harris L. K. Mccormick J. Cartwright J. E. Whitley G. S. Dash P. R. (2008). S-nitrosylation of proteins at the leading edge of migrating trophoblasts by inducible nitric oxide synthase promotes trophoblast invasion. *Exp. Cell Res.* 314 1765–1776 10.1016/j.yexcr.2008.02.010 18394602

[B53] Harwood B. N. Cross S. K. Radford E. E. Haac B. E De Vries W. N. (2008). Members of the WNT signaling pathways are widely expressed in mouse ovaries, oocytes, and cleavage stage embryos. *Dev. Dyn.* 237 1099–1111 10.1002/dvdy.21491 18351675

[B54] Hayashi K. Erikson D. W. Tilford S. A. Bany B. M. Maclean J. A. II, Rucker E. B. III (2009). Wnt genes in the mouse uterus: potential regulation of implantation. *Biol. Reprod.* 80 989–1000 10.1095/biolreprod.108.075416 19164167 PMC2804842

[B55] He S. Pant D. Schiffmacher A. Meece A. Keefer C. L. (2008). Lymphoid enhancer factor 1-mediated Wnt signaling promotes the initiation of trophoblast lineage differentiation in mouse embryonic stem cells. *Stem Cells* 26 842–849 10.1634/stemcells.2007-0356 18192238

[B56] Hendrickx M. Leyns L. (2008). Non-conventional Frizzled ligands and Wnt receptors. *Dev. Growth Differ.* 50 229–243 10.1111/j.1440-169X.2008.01016.x 18366384

[B57] Herr P. Hausmann G. Basler K. (2012). WNT secretion and signalling in human disease. *Trends Mol. Med.* 18 483–493 10.1016/j.molmed.2012.06.008 22796206

[B58] Hess A. P. Hamilton A. E. Talbi S. Dosiou C. Nyegaard M. Nayak N. (2007). Decidual stromal cell response to paracrine signals from the trophoblast: amplification of immune and angiogenic modulators. *Biol. Reprod.* 76 102–117 10.1095/biolreprod.106.054791 17021345

[B59] Hiby S. E. Walker J. J. O’Shaughnessy K M. Redman C. W. Carrington M. Trowsdale J. (2004). Combinations of maternal KIR and fetal HLA-C genes influence the risk of preeclampsia and reproductive success. *J. Exp. Med.* 200 957–965 10.1084/jem.20041214 15477349 PMC2211839

[B60] Hunkapiller N. M. Gasperowicz M. Kapidzic M. Plaks V. Maltepe E. Kitajewski J. (2011). A role for Notch signaling in trophoblast endovascular invasion and in the pathogenesis of pre-eclampsia. *Development* 138 2987–2998 10.1242/dev.066589 21693515 PMC3119307

[B61] Hustin J. Jauniaux E. Schaaps J. P. (1990). Histological study of the materno-embryonic interface in spontaneous abortion. *Placenta* 11 477–486 10.1016/S0143-4004(05)80193-6 2290799

[B62] Ishikawa T. Tamai Y. Zorn A. M. Yoshida H. Seldin M. F. Nishikawa S. (2001). Mouse Wnt receptor gene Fzd5 is essential for yolk sac and placental angiogenesis. *Development* 128 25–33 11092808 10.1242/dev.128.1.25

[B63] Ishitani T. Ishitani S. (2013). Nemo-like kinase, a multifaceted cell signaling regulator. *Cell. Signal.* 25 190–197 10.1016/j.cellsig.2012.09.017 23000342

[B64] Ishitani T. Ninomiya-Tsuji J. Nagai S. Nishita M. Meneghini M. Barker N. (1999). The TAK1-NLK-MAPK-related pathway antagonizes signalling between beta-catenin and transcription factor TCF. *Nature* 399 798–802 10.1038/21674 10391247

[B65] Janatpour M. J. Mcmaster M. T. Genbacev O. Zhou Y. Dong J. Cross J. C. (2000). Id-2 regulates critical aspects of human cytotrophoblast differentiation, invasion and migration. *Development* 127 549–558 10631176 10.1242/dev.127.3.549

[B66] Jokhi P. P. King A. Loke Y. W. (1994). Reciprocal expression of epidermal growth factor receptor (EGF-R) and c-erbB2 by non-invasive and invasive human trophoblast populations. *Cytokine* 6 433–442 10.1016/1043-4666(94)90068-X 7948752

[B67] Kim S. E. Lee W. J. Choi K. Y. (2007). The PI3 kinase-Akt pathway mediates Wnt3a-induced proliferation. *Cell. Signal.* 19 511–518 10.1016/j.cellsig.2006.08.008 17011750

[B68] Knöfler M. (2010). Critical growth factors and signalling pathways controlling human trophoblast invasion. *Int. J. Dev. Biol.* 54 269–280 10.1387/ijdb.082769mk 19876833 PMC2974212

[B69] Knöfler M. Pollheimer J. (2012). IFPA Award in Placentology lecture: molecular regulation of human trophoblast invasion. *Placenta* 33(Suppl.) S55–S62 10.1016/j.placenta.2011.09.019 22019198 PMC3272142

[B70] Kohn A. D. Moon R. T. (2005). Wnt and calcium signaling: beta-catenin-independent pathways. *Cell Calcium* 38 439–446 10.1016/j.ceca.2005.06.022 16099039

[B71] Komiya Y. Habas R. (2008). Wnt signal transduction pathways. *Organogenesis* 4 68–75 10.4161/org.4.2.5851 19279717 PMC2634250

[B72] Lala P. K. Chakraborty C. (2003). Factors regulating trophoblast migration and invasiveness: possible derangements contributing to pre-eclampsia and fetal injury. *Placenta* 24 575–587 10.1016/S0143-4004(03)00063-8 12828917

[B73] Lala P. K. Graham C. H. (1990). Mechanisms of trophoblast invasiveness and their control: the role of proteases and protease inhibitors. *Cancer* Metastasis Rev. 9 369–379 10.1007/BF00049525 2097085

[B74] Li Q. Kannan A. Das A. Demayo F. J. Hornsby P. J. Young S. L. (2013). WNT4 acts downstream of BMP2 and functions via beta-catenin signaling pathway to regulate human endometrial stromal cell differentiation. *Endocrinology* 154 446–457 10.1210/en.2012-1585 23142810 PMC3529366

[B75] Lim K. H. Zhou Y. Janatpour M. Mcmaster M. Bass K. Chun S. H. (1997). Human cytotrophoblast differentiation/invasion is abnormal in pre-eclampsia. *Am. J. Pathol.* 151 1809–1818 9403732 PMC1858365

[B76] Liu J. Wang H. Zuo Y. Farmer S. R. (2006). Functional interaction between peroxisome proliferator-activated receptor gamma and beta-catenin. *Mol. Cell. Biol.* 26 5827–5837 10.1128/MCB.00441-06 16847334 PMC1592783

[B77] Liu Y. Kodithuwakku S. P. Ng P. Y. Chai J. Ng E. H. Yeung W. S. (2010). Excessive ovarian stimulation up-regulates the Wnt-signaling molecule DKK1 in human endometrium and may affect implantation: an in vitro co-culture study. *Hum. Reprod.* 25 479–490 10.1093/humrep/dep429 19955106

[B78] Logan C. Y. Nusse R. (2004). The Wnt signaling pathway in development and disease. *Annu. Rev. Cell Dev. Biol.* 20 781–810 10.1146/annurev.cellbio.20.010403.113126 15473860

[B79] Loregger T. Pollheimer J. Knöfler M. (2003). Regulatory transcription factors controlling function and differentiation of human trophoblast–a review. *Placenta *24(Suppl. A) S104–S110 10.1053/plac.2002.0929 12842421

[B80] Lu J. Zhang S. Nakano H. Simmons D. G. Wang S. Kong S. (2013). A positive feedback loop involving Gcm1 and Fzd5 directs chorionic branching morphogenesis in the placenta. *PLoS Biol.* 11:e1001536 10.1371/journal.pbio.1001536 PMC3627642 23610556

[B81] Ma L. Wang H. Y. (2006). Suppression of cyclic GMP-dependent protein kinase is essential to the Wnt/cGMP/Ca2+ pathway. *J. Biol. Chem.* 281 30990–31001 10.1074/jbc.M603603200 16920709

[B82] Mao J. Wang J. Liu B. Pan W. Farr G. H. III Flynn C. (2001). Low-density lipoprotein receptor-related protein-5 binds to Axin and regulates the canonical Wnt signaling pathway. *Mol. Cell* 7 801–809 10.1016/S1097-2765(01)00224-6 11336703

[B83] Marchand M. Horcajadas J. A. Esteban F. J. Mcelroy S. L. Fisher S. J. Giudice L. C. (2011). Transcriptomic signature of trophoblast differentiation in a human embryonic stem cell model. *Biol. Reprod.* 84 1258–1271 10.1095/biolreprod.110.086413 21368299

[B84] Matsuura K. Jigami T. Taniue K. Morishita Y. Adachi S. Senda T. (2011). Identification of a link between Wnt/beta-catenin signalling and the cell fusion pathway. *Nat. Commun.* 2 548 10.1038/ncomms1551 22109522

[B85] Medici D. Hay E. D. Olsen B. R. (2008). Snail and Slug promote epithelial-mesenchymal transition through beta-catenin-T-cell factor-4-dependent expression of transforming growth factor-beta3. *Mol. Biol. Cell* 19 4875–4887 10.1091/mbc.E08-05-0506 18799618 PMC2575183

[B86] Mericskay M. Kitajewski J. Sassoon D. (2004). Wnt5a is required for proper epithelial-mesenchymal interactions in the uterus. *Development* 131 2061–2072 10.1242/dev.01090 15073149

[B87] Metcalfe C. Bienz M. (2011). Inhibition of GSK3 by Wnt signalling – two contrasting models. *J. Cell Sci.* 124 3537–3544 10.1242/jcs.091991 22083140

[B88] Mikels A. J. Nusse R. (2006). Purified Wnt5a protein activates or inhibits beta-catenin-TCF signaling depending on receptor context. *PLoS Biol.* 4:e115 10.1371/journal.pbio.0040115 PMC1420652 16602827

[B89] Miller C. Sassoon D. A. (1998). Wnt-7a maintains appropriate uterine patterning during the development of the mouse female reproductive tract. *Development* 125 3201–3211 9671592 10.1242/dev.125.16.3201

[B90] Minami Y. Oishi I. Endo M. Nishita M. (2010). Ror-family receptor tyrosine kinases in noncanonical Wnt signaling: their implications in developmental morphogenesis and human diseases. *Dev. Dyn.* 239 1–15 19530173 10.1002/dvdy.21991

[B91] Mohamed O. A. Dufort D. Clarke H. J. (2004). Expression and estradiol regulation of Wnt genes in the mouse blastocyst identify a candidate pathway for embryo-maternal signaling at implantation. *Biol. Reprod.* 71 417–424 10.1095/biolreprod.103.025692 15044261

[B92] Mohamed O. A. Jonnaert M. Labelle-Dumais C. Kuroda K. Clarke H. J. Dufort D. (2005). Uterine Wnt/beta-catenin signaling is required for implantation. *Proc. Natl. Acad. Sci. U.S.A.* 102 8579–8584 10.1073/pnas.0500612102 15930138 PMC1150820

[B93] Monkley S. J. Delaney S. J. Pennisi D. J. Christiansen J. H. Wainwright B. J. (1996). Targeted disruption of the Wnt2 gene results in placentation defects. *Development* 122 3343–3353 8951051 10.1242/dev.122.11.3343

[B94] Morrish D. W. Dakour J. Li H. (1998). Functional regulation of human trophoblast differentiation. *J. Reprod. Immunol.* 39 179–195 10.1016/S0165-0378(98)00021-7 9786461

[B95] Moustakas A. Heldin C. H. (2007). Signaling networks guiding epithelial-mesenchymal transitions during embryogenesis and cancer progression. *Cancer Sci.* 98 1512–1520 10.1111/j.1349-7006.2007.00550.x 17645776 PMC11158989

[B96] Nadeem L. Munir S. Fu G. Dunk C. Baczyk D. Caniggia I. (2011). Nodal signals through activin receptor-like kinase 7 to inhibit trophoblast migration and invasion: implication in the pathogenesis of preeclampsia. *Am. J. Pathol.* 178 1177–1189 10.1016/j.ajpath.2010.11.066 21356369 PMC3069932

[B97] Naito A. T. Akazawa H. Takano H. Minamino T. Nagai T. Aburatani H. (2005). Phosphatidylinositol 3-kinase-Akt pathway plays a critical role in early cardiomyogenesis by regulating canonical Wnt signaling. *Circ. Res.* 97 144–151 10.1161/01.RES.0000175241.92285.f8 15994435

[B98] Nishita M. Enomoto M. Yamagata K. Minami Y. (2010). Cell/tissue-tropic functions of Wnt5a signaling in normal and cancer cells. *Trends Cell Biol.* 20 346–354 10.1016/j.tcb.2010.03.001 20359892

[B99] Novakovic B. Rakyan V. Ng H. K. Manuelpillai U. Dewi C. Wong N. C. (2008). Specific tumour-associated methylation in normal human term placenta and first-trimester cytotrophoblasts. *Mol. Hum. Reprod.* 14 547–554 10.1093/molehr/gan046 18708652

[B100] Nusse R. Brown A. Papkoff J. Scambler P. Shackleford G. Mcmahon A. (1991). A new nomenclature for int-1 and related genes: the Wnt gene family. *Cell* 64 231 10.1016/0092-8674(91)90633-A 1846319

[B101] Oreshkova T. Dimitrov R. Mourdjeva M. (2012). A cross-talk of decidual stromal cells, trophoblast, and immune cells: a prerequisite for the success of pregnancy. *Am. J. Reprod. Immunol.* 68 366–373 10.1111/j.1600-0897.2012.01165.x 22672047

[B102] Parr B. A. Cornish V. A. Cybulsky M. I. Mcmahon A. P. (2001). Wnt7b regulates placental development in mice. *Dev. Biol.* 237 324–332 10.1006/dbio.2001.0373 11543617

[B103] Peng S. Li J. Miao C. Jia L. Hu Z. Zhao P. (2008). Dickkopf-1 secreted by decidual cells promotes trophoblast cell invasion during murine placentation. *Reproduction* 135 367–375 10.1530/REP-07-0191 18299430

[B104] Peng S. Miao C. Li J. Fan X. Cao Y. Duan E. (2006). Dickkopf-1 induced apoptosis in human placental choriocarcinoma is independent of canonical Wnt signaling. *Biochem. Biophys. Res. Commun.* 350 641–647 10.1016/j.bbrc.2006.09.087 17026960

[B105] Pijnenborg R. Anthony J. Davey D. A. Rees A. Tiltman A. Vercruysse L. (1991). Placental bed spiral arteries in the hypertensive disorders of pregnancy. *Br. J. Obstet. Gynaecol.* 98 648–655 10.1111/j.1471-0528.1991.tb13450.x 1883787

[B106] Pijnenborg R. Vercruysse L. Hanssens M. (2006). The uterine spiral arteries in human pregnancy: facts and controversies. *Placenta* 27 939–958 10.1016/j.placenta.2005.12.006 16490251

[B107] Poehlmann T. G. Fitzgerald J. S. Meissner A. Wengenmayer T. Schleussner E. Friedrich K. (2005). Trophoblast invasion: tuning through LIF, signalling via Stat3. *Placenta *26(Suppl. A) S37–S41 10.1016/j.placenta.2005.01.007 15837065

[B108] Polakis P. (2000). Wnt signaling and cancer. *Genes Dev.* 14 1837–1851 10921899

[B109] Pollheimer J. Bauer S. Huber A. Husslein P. Aplin J. D. Knöfler M. (2004). Expression pattern of collagen XVIII and its cleavage product, the angiogenesis inhibitor endostatin, at the fetal-maternal interface. *Placenta* 25 770–779 10.1016/j.placenta.2004.03.003 15451191

[B110] Pollheimer J. Haslinger P. Fock V. Prast J. Saleh L. Biadasiewicz K. (2011). Endostatin suppresses IGF-II-mediated signaling and invasion of human extravillous trophoblasts. *Endocrinology* 152 4431–4442 10.1210/en.2011-1196 21933871

[B111] Pollheimer J. Husslein P. Knöfler M. (2005). Invasive trophoblasts generate regulatory collagen XVIII cleavage products. *Placenta* 26(Suppl. A) S42–S45 10.1016/j.placenta.2004.12.005 15837066

[B112] Pollheimer J. Knöfler M. (2005). Signalling pathways regulating the invasive differentiation of human trophoblasts: a review. *Placenta *26(Suppl. A) S21–S30 10.1016/j.placenta.2004.11.013 15837062

[B113] Pollheimer J. Loregger T. Sonderegger S. Saleh L. Bauer S. Bilban M. (2006). Activation of the canonical wingless/T-cell factor signaling pathway promotes invasive differentiation of human trophoblast. *Am. J. Pathol.* 168 1134–1147 10.2353/ajpath.2006.050686 16565489 PMC1606554

[B114] Prakobphol A. Genbacev O. Gormley, M., Kapidzic M. Fisher S. J. (2006). A role for the L-selectin adhesion system in mediating cytotrophoblast emigration from the placenta. *Dev. Biol.* 298 107–117 10.1016/j.ydbio.2006.06.020 16930583

[B115] Rauwel B. Mariame B. Martin H. Nielsen R. Allart S. Pipy B. (2010). Activation of peroxisome proliferator-activated receptor gamma by human cytomegalovirus for de novo replication impairs migration and invasiveness of cytotrophoblasts from early placentas. *J. Virol.* 84 2946–2954 10.1128/JVI.01779-09 20042507 PMC2826069

[B116] Red-Horse K. Zhou Y. Genbacev O. Prakobphol A. Foulk R. Mcmaster M. (2004). Trophoblast differentiation during embryo implantation and formation of the maternal-fetal interface. *J. Clin. Invest.* 114 744–754 15372095 10.1172/JCI22991 PMC516273

[B117] Redman C. W. Sargent I. L. (2010). Immunology of pre-eclampsia. *Am. J. Reprod. Immunol.* 63 534–543 10.1111/j.1600-0897.2010.00831.x 20331588

[B118] Redman C. W. Tannetta D. S. Dragovic R. A. Gardiner C. Southcombe J. H. Collett G. P. (2012). Review: does size matter? Placental debris and the pathophysiology of pre-eclampsia. *Placenta* 33(Suppl.) S48–S54 10.1016/j.placenta.2011.12.006 22217911

[B119] Rena V. Angeletti S. Panzetta-Dutari G. Genti-Raimondi S. (2009). Activation of beta-catenin signalling increases StarD7 gene expression in JEG-3 cells. *Placenta* 30 876–883 10.1016/j.placenta.2009.07.010 19679347

[B120] Robson A. Harris L. K. Innes B. A. Lash G. E. Aljunaidy M. M. Aplin J. D. (2012). Uterine natural killer cells initiate spiral artery remodeling in human pregnancy. *FASEB J.* 26 4876–4885 10.1096/fj.12-210310 22919072

[B121] Roose J. Clevers H. (1999). TCF transcription factors: molecular switches in carcinogenesis. *Biochim. Biophys. Acta* 1424 M23–M37 10528152 10.1016/s0304-419x(99)00026-8

[B122] Sanchez-Tillo E. De Barrios O. Siles L. Cuatrecasas M. Castells A. Postigo A. (2011). beta-catenin/ TCF4 complex induces the epithelial-to-mesenchymal transition (EMT)-activator ZEB1 to regulate tumor invasiveness. *Proc. Natl. Acad. Sci. U.S.A.* 108 19204–19209 10.1073/pnas.1108977108 22080605 PMC3228467

[B123] Saneyoshi T. Kume S. Amasaki Y. Mikoshiba K. (2002). The Wnt/calcium pathway activates NF-AT and promotes ventral cell fate in Xenopus embryos. *Nature* 417 295–299 10.1038/417295a 12015605

[B124] Sonderegger S. Haslinger P. Sabri A. Leisser C. Otten J. V. Fiala C. (2010a). Wingless (Wnt)-3A induces trophoblast migration and matrix metalloproteinase-2 secretion through canonical Wnt signaling and protein kinase B/AKT activation. *Endocrinology* 151 211–220 10.1210/en.2009-0557 19887570 PMC2974214

[B125] Sonderegger S. Husslein H. Leisser C. Knöfler M. (2007). Complex expression pattern of Wnt ligands and frizzled receptors in human placenta and its trophoblast subtypes. *Placenta *28(Suppl. A) S97–S102 10.1016/j.placenta.2006.11.003 PMC2963058 17198728

[B126] Sonderegger S. Pollheimer J. Knöfler M. (2010b). Wnt signalling in implantation, decidualisation and placental differentiation – review. *Placenta* 31 839–847 10.1016/j.placenta.2010.07.011 20716463 PMC2963059

[B127] Stamos J. L. Weis W. I. (2013). The beta-catenin destruction complex. *Cold Spring Harb. Perspect. Biol.* 5 a007898 10.1101/cshperspect.a007898 PMC3579403 23169527

[B128] Tal R. (2012). The role of hypoxia and hypoxia-inducible factor-1alpha in preeclampsia pathogenesis. *Biol. Reprod.* 87 134 10.1095/biolreprod.112.102723 23034156

[B129] Tsang H. Cheung T. Y. Kodithuwakku S. P. Chai J. Yeung W. S. Wong C. K. (2012). 2,3,7,8-Tetrachlorodibenzo-p-dioxin (TCDD) suppresses spheroids attachment on endometrial epithelial cells through the down-regulation of the Wnt-signaling pathway. *Reprod. Toxicol.* 33 60–66 10.1016/j.reprotox.2011.11.002 22134133

[B130] Tulac S. Nayak N. R. Kao L. C. Van Waes M. Huang J. Lobo S. (2003). Identification, characterization, and regulation of the canonical Wnt signaling pathway in human endometrium. *J. Clin. Endocrinol. Metab.* 88 3860–3866 10.1210/jc.2003-030494 12915680

[B131] Tulac S. Overgaard M. T. Hamilton A. E. Jumbe N. L. Suchanek E. Giudice L. C. (2006). Dickkopf-1, an inhibitor of Wnt signaling, is regulated by progesterone in human endometrial stromal cells. *J. Clin. Endocrinol. Metab.* 91 1453–1461 10.1210/jc.2005-0769 16449346

[B132] Vainio S. Heikkila M. Kispert A. Chin N. Mcmahon A. P. (1999). Female development in mammals is regulated by Wnt-4 signalling. *Nature* 397 405–409 10.1038/17068 9989404

[B133] van Amerongen R. Nusse R. (2009). Towards an integrated view of Wnt signaling in development. *Development* 136 3205–3214 10.1242/dev.033910 19736321

[B134] van der Horst P. H. Wang Y. Van Der Zee M. Burger C. W. Blok L. J. (2012). Interaction between sex hormones and WNT/beta-catenin signal transduction in endometrial physiology and disease. *Mol. Cell. Endocrinol.* 358 176–184 10.1016/j.mce.2011.06.010 21722706

[B135] van Dijk M. Oudejans C. B. (2011). STOX1: key player in trophoblast dysfunction underlying early onset preeclampsia with growth retardation. *J. Pregnancy* 2011 521826 10.1155/2011/521826 PMC3066643 21490791

[B136] Wang Q. Lu J. Zhang S. Wang S. Wang W. Wang B. (2013). Wnt6 is essential for stromal cell proliferation during decidualization in mice. *Biol. Reprod.* 88 5 10.1095/biolreprod.112.104687 23175771

[B137] Wang Y. (2009). Wnt/planar cell polarity signaling: a new paradigm for cancer therapy. *Mol. Cancer Ther.* 8 2103–2109 10.1158/1535-7163.MCT-09-0282 19671746

[B138] Wetendorf M. DeMayo F. J. (2012). The progesterone receptor regulates implantation, decidualization, and glandular development via a complex paracrine signaling network. *Mol. Cell. Endocrinol.* 357 108–118 10.1016/j.mce.2011.10.028 22115959 PMC3443857

[B139] Wodarz A. Nusse R. (1998). Mechanisms of Wnt signaling in development. *Annu. Rev. Cell Dev. Biol.* 14 59–88 10.1146/annurev.cellbio.14.1.59 9891778

[B140] Wong N. C. Novakovic B. Weinrich B. Dewi C. Andronikos R. Sibson M. (2008). Methylation of the adenomatous polyposis coli (APC) gene in human placenta and hypermethylation in choriocarcinoma cells. *Cancer Lett.* 268 56–62 10.1016/j.canlet.2008.03.033 18485586

[B141] Wu B. Crampton S. P. Hughes C. C. (2007). Wnt signaling induces matrix metalloproteinase expression and regulates T cell transmigration. *Immunity* 26 227–239 10.1016/j.immuni.2006.12.007 17306568 PMC1855210

[B142] Wu Q. L. Zierold C. Ranheim E. A. (2009). Dysregulation of Frizzled 6 is a critical component of B-cell leukemogenesis in a mouse model of chronic lymphocytic leukemia. *Blood* 113 3031–3039 10.1182/blood-2008-06-163303 19179304 PMC2945928

[B143] Xie H. Tranguch S. Jia X. Zhang H. Das S. K. Dey S. K. (2008). Inactivation of nuclear Wnt-beta-catenin signaling limits blastocyst competency for implantation. *Development* 135 717–727 10.1242/dev.015339 18199579 PMC2829274

[B144] Yun M. S. Kim S. E. Jeon S. H. Lee J. S. Choi K. Y. (2005). Both ERK and Wnt/beta-catenin pathways are involved in Wnt3a-induced proliferation. *J. Cell Sci.* 118 313–322 10.1242/jcs.01601 15615777

[B145] Zhang Z. Li H. Zhang L. Jia L. Wang P. (2013a). Differential expression of beta-catenin and dickkopf-1 in the third trimester placentas from normal and preeclamptic pregnancies: a comparative study. *Reprod. Biol. Endocrinol.* 11 17 10.1186/1477-7827-11-17 PMC3599361 23452984

[B146] Zhang Z. Zhang L. Jia L. Wang P. Gao Y. (2013b). Association of Wnt2 and sFRP4 expression in the third trimester placenta in women with severe preeclampsia. *Reprod. Sci*. 20 981–989 10.1177/1933719112472740 23322712 PMC3713648

[B147] Zheng H. Kang Y. (2013). Multilayer control of the EMT master regulators. *Oncogene*. 10.1038/onc.2013.128 [Epub ahead of print]. 23604123

[B148] Zhou Y. Damsky C. H. Fisher S. J. (1997a). Preeclampsia is associated with failure of human cytotrophoblasts to mimic a vascular adhesion phenotype. One cause of defective endovascular invasion in this syndrome?* J. Clin. Invest.* 99 2152–2164 10.1172/JCI119388 PMC508045 9151787

[B149] Zhou Y. Fisher S. J. Janatpour M. Genbacev O. Dejana E. Wheelock M. (1997b). Human cytotrophoblasts adopt a vascular phenotype as they differentiate. A strategy for successful endovascular invasion? *J. Clin. Invest.* 99 2139–2151 10.1172/JCI119387 9151786 PMC508044

[B150] Zhou Y. Gormley M. J. Hunkapiller N. M. Kapidzic M. Stolyarov Y. Feng V. (2013). Reversal of gene dysregulation in cultured cytotrophoblasts reveals possible causes of preeclampsia. *J. Clin. Invest.* 123 2862–2872 10.1172/JCI66966 23934129 PMC3999620

